# Triple Generative Self-Supervised Learning Method for Molecular Property Prediction

**DOI:** 10.3390/ijms25073794

**Published:** 2024-03-28

**Authors:** Lei Xu, Leiming Xia, Shourun Pan, Zhen Li

**Affiliations:** College of Computer Science and Technology, Qingdao University, Qingdao 266071, China; 2021023807@qdu.edu.cn (L.X.); 2021020690@qdu.edu.cn (L.X.); 2021020685@qdu.edu.cn (S.P.)

**Keywords:** generative supervised learning, variational auto-encoders, molecular feature extraction, molecular property prediction, artificial intelligence

## Abstract

Molecular property prediction is an important task in drug discovery, and with help of self-supervised learning methods, the performance of molecular property prediction could be improved by utilizing large-scale unlabeled dataset. In this paper, we propose a triple generative self-supervised learning method for molecular property prediction, called TGSS. Three encoders including a bi-directional long short-term memory recurrent neural network (BiLSTM), a Transformer, and a graph attention network (GAT) are used in pre-training the model using molecular sequence and graph structure data to extract molecular features. The variational auto encoder (VAE) is used for reconstructing features from the three models. In the downstream task, in order to balance the information between different molecular features, a feature fusion module is added to assign different weights to each feature. In addition, to improve the interpretability of the model, atomic similarity heat maps were introduced to demonstrate the effectiveness and rationality of molecular feature extraction. We demonstrate the accuracy of the proposed method on chemical and biological benchmark datasets by comparative experiments.

## 1. Introduction

Drug development is a time-consuming and costly process. In order to improve the success rate and reduce the time and costs, computer-aided drug design (CADD) [1,2] methods such as virtual screening and molecular docking have been introduced to provide guidance for the entire process. Despite their success in drug discovery [3,4], many traditional CADD methods based on molecular simulation techniques suffer from high computational costs and long running times, which limit their large-scale application in the pharmaceutical industry.

In recent years, artificial intelligence has developed rapidly, which has become a popular and dominant direction within drug discovery because of its superior performance and high efficiency. Moreover, many deep-learning methods [5,6] have been successfully applied to various tasks in drug discovery, including molecular property prediction [7], drug-target affinity prediction [8,9], and protein–protein interaction prediction [10].

Molecular property prediction aims to predict whether a molecule has the expected properties (solubility, biological activity, etc.) from a large number of candidate molecules, which is important for drug design. There are many ways to represent molecular sequences, including simplified molecular input line entry systems (SMILES), and fingerprints like Extended-Connectivity fingerprints (ECFP) [11] and the molecular access system (MACCS) [12]. SMILES is a specification for extracting molecular sequence features that uses ASCII strings to encode molecular structures. The molecule is simply represented using one or two letter symbols from the periodic table. For chemical bonds [13], single bonds can be implicitly represented by “-”, and double, triple, and quadruple bonds are represented by “=”, “#”, and “$”, respectively. Various deep learning methods based on SMILES strings have emerged. Hou et al. [14] used LSTM to process SMILES strings to obtain complex information of atoms. Honda et al. [15] proposed the SMILES Transformer, which pre-trained the sequence-to-sequence model by using SMILES strings. However, the structural information of molecules cannot be obtained from a SMILES string directly since two connected atoms may be far away from each other in the SMILES string.

Moreover, the molecular graph structure [16] provided another way to represent molecules, in which atoms are represented by nodes and chemical bonds are represented by edges. In recent years, many studies have concentrated on molecular graph structures for molecular property prediction through Message Passing Networks, including MPNN [17], DMPNN [18], and CMPNN [19]. Most of current graph-based methods are supervised learning methods that require large-scale labeled data for training. However, the label acquirement (i.e., molecules with known property) is a tough and expensive process. On the other hand, there are many databases with a large amount of data but no label information (e.g., ZINC [20], ChEMBL, and PubChem). How to reasonably and effectively utilize these data to improve the accuracy of molecular property prediction is an open problem to be solved.

The Natural Language Processing (NLP) and Computer Vision [21] (CV) fields address this problem through self-supervised learning (SSL). Specifically, the model is first pre-trained on a large unlabeled dataset and then fine-tuned for downstream tasks using data with labels. SSL includes generative self-supervised and contrastive self-supervised learning [22]. The generative SSL consists of an encoder and a decoder. The encoder is trained to encode an input x into a latent vector z, and the decoder is used to reconstruct z into x by minimizing the reconstruction loss. The generative SSL methods include AutoRegressive (AR) models, flow-based models, AutoEncoder (AE) models, and hybrid generative models. For the contrastive SSL [23,24], features are learned by constructing positive and negative samples, and an encoder is trained to encode an input x into an explicit vector z to measure similarity.

SSL has achieved great success in the field of natural language processing, such as the creation of the Generative Pre-trained Transformer (GPT) [25] and Bidirectional Encoder Representation from Transformers (BERT) [26]. GPT is OpenAI’ s pre-trained Transformer model for natural language processing, which uses deep learning to generate human language-like text given a prompt or seed text. The pre-trained BERT language model is able to learn contextual word representations by masking words prediction and reconstructing the input context, thereby improving the performance of downstream tasks. Wen et al. [27] pre-trained BERT to obtain a semantic representation of compound fingerprints through SSL, called Fingerprint-BERT (FP-BERT). Then, the embedding of molecule was fed into a convolutional neural network (CNN) to obtain higher-level features.

However, language models can only be used to handle sequence-based molecular representations, ignoring the important topology of molecular graphs. Therefore, the utilization of SSL for molecular graphs is also a non-negligible aspect of molecular property prediction. Graph contrastive coding (GCC) [28] designs a self-supervised graph neural network pre-training framework to capture common network topological properties across multiple networks. The KPGT [29] self-supervised framework introduces the line graph transformer (LiGhT), which is mainly used to accurately simulate the structural information of molecular graphs. However, it ignores the unique structural properties of chemical molecules, such as rings and functional groups. To fully consider the properties of molecular graphs, Zhang et al. [30] sampled subgraphs by learning graph motifs. The motif learning was defined as a clustering problem through EM-clustering to group similar and important subgraphs into several motifs. These learned motifs were used to train the sampler to generate more informative subgraphs for graph-to-subgraph contrastive learning. HiMol [31] used a hierarchical molecular graph neural network (HMGNN) to encode topic structures and extracted node–topic–graph hierarchical molecular representations.

Despite improvements in molecular representation learning, there are still some problems which remain to be solved:

(1) Although molecular representations based on SSL have been extensively studied, most methods focus on pre-training using sequence information or graph information only. The effective fusion of heterogeneous molecular information is important for enhancing the diversity of molecular representations. There are some methods that have considered this direction. Liu et al. [32] used 3D and 2D information for SSL, aiming to maximize the mutual information between 3D and 2D views of the same molecule. However, there is much less 3D molecular structural information than there is 2D and 1D information. Although there are some methods that could calculate 3D information about a molecule, the error accumulation could also result in the inaccuracy of predictions. Zhu et al. [33] used sequence and graph information to conduct SSL and proposed a pre-training algorithm that combined two molecular representations, including dual-view molecular pre-training (DMP), which maximized the consistency between molecular sequence and molecular graph representations. However, we believe that the generative model can reflect molecular information more accurately and effectively. Therefore, inspired by Liu’s work, this paper concentrates on how to use the generative SSL model to learn molecular representations from sequence and topological structural information from molecules.
(2) The existing SSL models, whether generative or contrastive, generally only use a single or two different models. For example, in generative learning, the encoder and decoder are used to reconstruct features, and in contrastive learning, SSL is performed by minimizing the difference between the feature representation of two different types or sources of data. But there is currently no method to discuss the introduction of three or more models in SSL. We believe that, to a certain extent, more models participating in SSL can also improve the accuracy and generalization of the final feature representation.
(3) After pre-training, multiple models are obtained for downstream tasks, and how to more effectively integrate multiple models is also a problem worth studying. Ensemble learning is widely used in the fusion of various models, but directly concatenating output features cannot effectively utilize the advantages of different models. Treating each output feature equally will also result in key information vanishing from multiple features. Therefore, how to design an effective fusion model, discover the important parts of different sources of features, and improve the accuracy of the prediction are also important issues in this paper.

To address the above problems, a triple generative self-supervised learning method (TGSS) is proposed in this paper, which combines molecular sequence information and molecular graph structure information to improve model performance. Moreover, BiLSTM and Transformer are used to learn the feature representation of the molecular sequence, and GAT is used to learn the feature representation of the molecular graph. The generative SSL method is introduced in the pre-training step and all three representations are used for reconstruction, which are performed in pairs to improve the generalization of the model. For the downstream tasks, all three pre-trained models are fused and the attention module is utilized to fully integrate the three features. We experimented with eight downstream tasks of molecular property prediction, five of which outperform existing supervised and self-supervised learning methods.

## 2. Results

### 2.1. Datasets

For the pre-training dataset, we used 430,000 unlabeled molecules randomly sampled from the public ChEMBL database available at https://www.ebi.ac.uk/chembl/ (accessed on 3 March 2023). ChEMBL is a database of bioactive molecules with drug-like properties, containing millions of unlabeled SMILES data. The comparative experiments were tested on the public dataset MoleculeNet [34] available at https://moleculenet.org/ (accessed on 5 April 2023), including classification tasks and regression tasks. For the regression task dataset, we use the random splitting method to divide the dataset. For the classification task dataset, following the method of Yang et al. [35], we use the scaffold splitting, which splits the molecules according to their structures. The molecular samples in the training set and the test set come from different molecular scaffolds. This scaffold splitting method is more challenging and could evaluate the generalization of model more accurately. These two split methods are used to split a dataset into a training set, validation set, and test set in the ratio of 8:1:1. The details of the dataset are shown in Table 1. It should be noted that Tox21 and SIDER involve multi-classification tasks, where each input sample may correspond to multiple labels. Therefore, the arithmetic mean values of all labels are calculated for these two datasets as the final result.

Regression dataset:

- FreeSolv: the experiment and calculated hydration-free energies in water of 642 small neutral molecules.
- ESOL: 1128 compounds and their corresponding water solubility.
- Lipophilicity: the octanol/water partition coefficients of 4200 compounds.
- Classification dataset.

Classification dataset:

- BACE: the quantitative and qualitative binding results for a panel of human (BACE-1) inhibitors.
- BBBP: the permeability properties of 2039 compounds.
- HIV: more than 40,000 compounds with the ability to inhibit HIV replication, represented by inactivated and active tags.
- Tox21: the qualitative toxicity measurements of 12 different targets for 7831 compounds.
- SIDER: 27 drug side effects labels for 1427 compounds.

### 2.2. Performance Comparison with Baselines

To better test the model performance, we have selected self-supervised learning models and supervised learning models for comparison. Self-supervised models includes MolCLR [36], GraphCL [37], HierMRL [38], GraphLoG [39], GraphMVP [32], GraphMAE [40], KEMPNN [41], MolPMoFiT [42], MolBERT [43], FP-BERT [27], and SMILES Transforme [15]. Supervised learning models includes D-MPNN [35], DimeNet [44], AttentionFP [45], DLF-MFF [46], and MSSGAT [47].

To demonstrate the effectiveness of the TGSS method, we tested it on eight molecular datasets, and the experimental results are shown in Table 2 and Table 3, which were obtained using the mean and standard deviation of three different random seed tests. Table 2 shows the performance of the TGSS method in classification tasks. Compared with other supervised/self-supervised learning methods, our TGSS method performed the best on BBBP, HIV, SIDER, in the five benchmark datasets in the classification tasks. Compared to the best results on these three datasets, the TGSS method achieved improvements of 5.7%, 0.3%, and 4.9%, respectively. Specifically, our TGSS achieved the best overall performance on five datasets compared to the supervised learning and other self-supervised learning methods, including generative and contrastive SSL models. These results demonstrate the effectiveness and good generalization ability of our self-supervised strategy.

Table 3 shows the performance of the TGSS method in regression tasks. It can be seen from the table that our TGSS method outperformed the previous supervised learning method on all three datasets. Compared with other self-supervised learning methods, although our method is weaker than MolBERT and KEMPNN on the ESOL and Lipophilicity datasets, respectively, the overall performance is better when combining the three datasets. It is worth noting that the improvement made by our TGSS method on the FreeSolv dataset was by 18.8%; thus, it can be seen that the improvement made by our model was most significant in small datasets. This effectively demonstrated that the TGSS model was capable of extracting effective representations from limited molecular data.

### 2.3. Ablation Experiments

To explore the influence of different factors on the model’s performance, we conducted ablation experiments in the pre-training, downstream task prediction, and feature fusion stages, respectively.

#### 2.3.1. Performance Comparison of Different Combinations of the Model in the Pre-Training Process

In this paper, three models including BiLSTM, Transformer, and GAT were embedded in the TGSS to improve the generalization performance of the model. To explore the impact of different pre-trained models, we designed the first ablation experiment with five groups: pre-training all three models, only pre-training two models, including BiLSTM and GAT (Pre-BG), BiLSTM and Transformer (Pre-BT), Transformer and GAT (Pre-TG), that is, $L_{xz}$, $L_{xy}$, $L_{yz}$ are used as the objective functions alone, and the last group is without pre-training (No pre). For a fair comparison, the experiment still used the three models’ fusion prediction methods in the downstream tasks, but the parameters of the models that did not participate in pre-training were initialized randomly.

**Figure 1:**
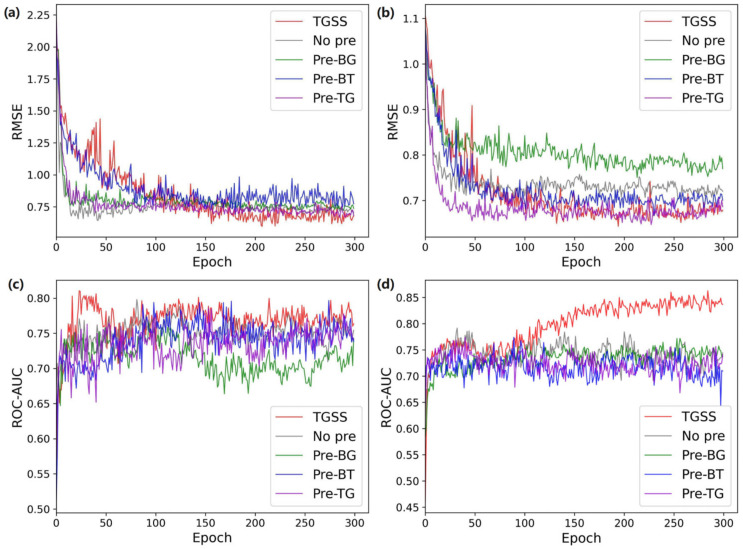
Performance comparison of different combinations of model in pre-training process. (**a**) ESOL. (**b**) Lipophilicity. (**c**) BACE. (**d**) BBBP.

Four datasets including two regression tasks (ESOL and Lipophilicity) and two classification tasks (BACE and BBBP) were selected for evaluation. The prediction result at different epochs was used as the indicator for different methods. From the ESOL dataset in Figure 1a it can be seen that, in the first 100 epochs, the effect of the proposed TGSS model was worse than Pre-BG, Pre-TG, and no pre-training. After the 100th epoch, the RMSE value became the minimum one. It could be concluded that pre-training has significantly improved the performance of the model. Compared with only pre-training BG, BT, TG, and no pre-training, the results were improved by 19.8%, 15.7%, 11.7% and 7.6%, respectively. Since the amount of data in Lipophilicity was larger than that in ESOL, after increasing the amount of data, the gap between each module widened. Pre-training with BiLSTM and Transformer, Transformer and GAT, and the proposed TGSS method were all significantly better than no pre-training. At the 195th epoch, the proposed model achieved the best results; compared with the best results of pre-BG, pre-BT, pre-TG, and no pre-training, the improvements were 17.2%, 3.7%, 0.6%, and 9.0%, respectively. In Figure 1b, it can be seen that the TGSS model achieved the best performance faster than other methods, and the curve is smoother, indicating that it has better stability. Therefore, it was found that pre-training the model for downstream tasks effectively improved the prediction accuracy. Compared with the non-pre-trained model, the pre-trained model achieved convergence faster, which sped up the training process.

#### 2.3.2. Performance Comparison of Different Sizes of Pre-Training Dataset

The model was pre-trained to learn effective molecular representations without labels through SSL. A sub-dataset which contains 430,000 molecules was used in pre-training. To explore whether less data would affect the performance of downstream tasks, we implemented the pre-training with different amounts of data, from 10,000 to 430,000, and tested its performance on downstream tasks.

**Figure 2:**
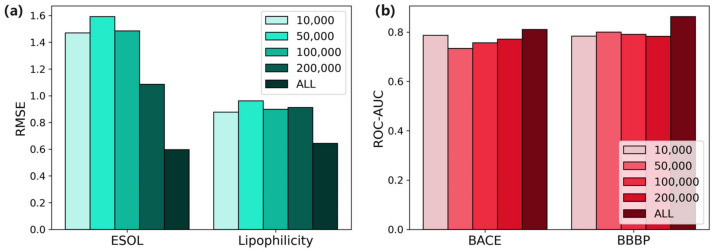
Performance comparison of different sizes of pre-training dataset. (**a**) Regression tasks. (**b**) Classification tasks.

It can be clearly seen from Figure 2a that, on the regression dataset, pre-training with more data effectively improved the performance of the model. The RMSE using the whole dataset was 0.597, whereas the RMSE using the pre-training dataset with 20,000 molecules was 1.086, which was clearly improved through the use of a larger dataset. For the classification tasks in Figure 2b, the improvements brought by using 430,000 molecules as the pre-training dataset compared to using other smaller datasets are clear. To summarize, the amount of pre-training data affected the performance. By increasing the amount of pre-training data, the model could learn more comprehensive molecular features, thereby improving the generalization ability of the model. 

#### 2.3.3. Performance Comparison of Different Combinations of Model in Downstream Tasks

**Figure 3:**
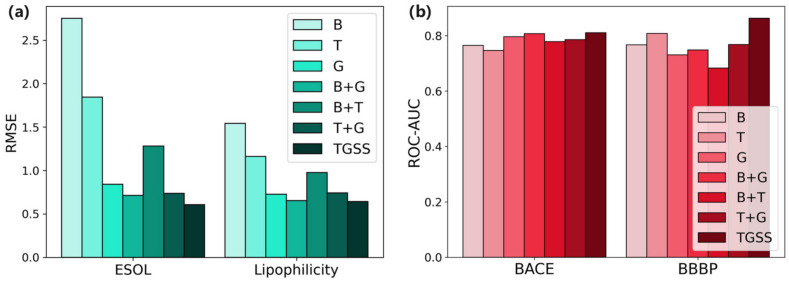
Performance comparison of different combinations of model in downstream tasks (**a**) Regression tasks. (**b**) Classification tasks.

In the downstream task, we used three models to predict molecular properties. Among them, BiLSTM and Transformer extracted molecular sequence features, and GAT extracted 2D molecular graph features. In this section, we try to investigate the contribution of each single model in a downstream task. For a fair comparison, all three trained models were acquired from the TGSS pre-training step. Instead of combining them together, each single model, BiLSTM (B), Transformer (T), and GAT (G), and the fusion of any two models (B + G, B + T, T + G) were used for comparison, and the results are shown in Figure 3.

As can be seen from Figure 3a, the improvement of our TGSS model is even more obvious on the ESOL dataset, which is about 18.3% compared with the other best combinations. When using the larger regression dataset, Lipophilicity, although the fusion of BiLSTM and GAT had achieved an RMES of 0.653, our method still led to an improvement of about 1.6%. As can be seen in Figure 3b, for the classification task, the proposed TGSS method led to an improvement of about 0.4% on the smaller BACE dataset and 6.4% on the BBBP dataset. Through the experiment, it was demonstrated that the proposed TGSS model combining three models could obtain the best results and improve the generalization performance of the model. This showed that using multiple models to learn molecular information was effective. Different models could learn various aspects of molecular information, thus compensating for the limitations of a single model, meaning that the proposed model could comprehensively acquire molecular information.

#### 2.3.4. Performance Comparison of Different Feature Fusion Methods

When merging different molecular features, we believe that concatenating two features directly cannot explore the deep information of each feature, and so we introduced aa hierarchical elem-feature fusion method to the TGSS model. In this section, we experimented with two different strategies for the model, direct concatenating and adding the hierarchical elem-feature fusion modules, to explore their different impacts on the model.

**Figure 4:**
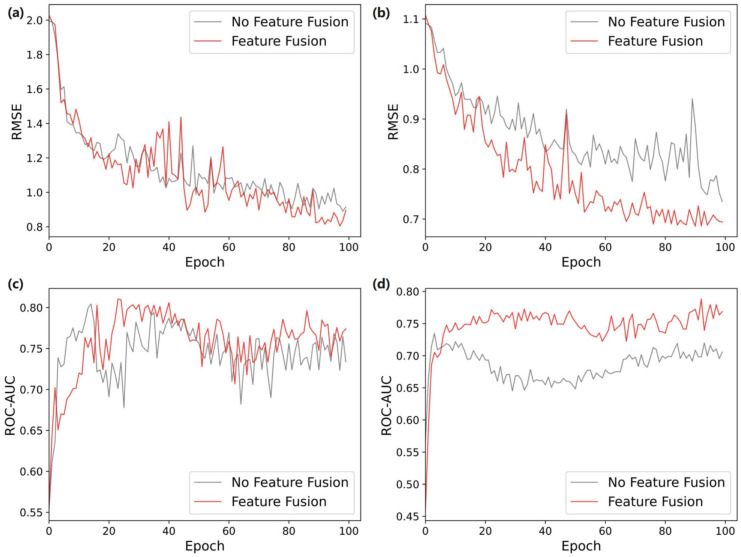
Performance comparison of different feature fusion methods. (**a**) ESOL. (**b**) Lipophilicity. (**c**) BACE. (**d**) BBBP.

For the three molecular features extracted by the model, adding a feature fusion method could effectively balance the proportion of the three in the final output features. As shown in Figure 4a, in the ESOL dataset, adding feature fusion could achieve a lower RMSE than no fusion method. It can be seen from Figure 4b that, on the larger dataset, Lipophilicity, the curves of the two were more consistent, but the improvement after adding feature fusion to the best result was about 8.6%. For the classification dataset, the feature fusion was able to significantly improve the prediction performance, and this trend is evident from Figure 4d. From the experiments, it was found that feature fusion could prevent the premature fitting of the model when the amount of data was small. Although the effect of the improvement was not as obvious as when the amount of data increased, it was still better than directly concatenating features.

### 2.4. Feature Visualization

TGSS has shown good results on various datasets, but there are still interpretability problems within the model. Due to the black-box nature of the deep learning model, the learned content (weights, features) cannot be effectively mapped to chemistry, biology, or other knowledge domains. Therefore, visualizing what the model has learned can help measure the effectiveness of the model and improve the interpretability of the model.

Molecular features consist of the individual features of each atom. For the TGSS model, there are three representations for each atomic feature, which are BiLSTM features, Transformer features, and GAT features. In order to study the evolution of these features during the training process, we calculated the similarity coefficient (Pearson correlation coefficient) between atomic features, and then visualized the similarity with a heat map.

**Figure 5:**
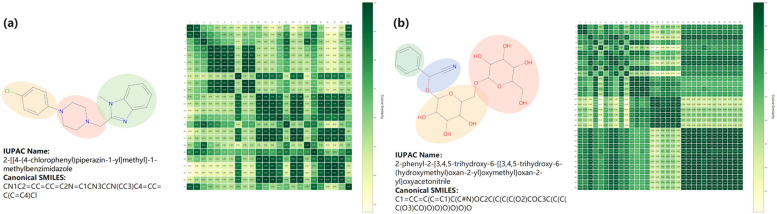
Atomic similarity heat map. (**a**) Example in the Lipophilicity dataset. (**b**) Example in the ESOL dataset.

We randomly selected a molecule from the datasets of Lipophilicity and ESOL for mapping, and plotted them as final output features. As can be seen from Figure 5a, the TGSS model combined with the three features’ information clearly showed the clustering of atoms. After 100 epochs, the molecules were divided into three clusters, namely 4-chlorophenyl, 1-methylbenzimidazole, and piperazine. Moreover, both 4-chlorophenyl and 1-methylbenzimidazole are lipophilic, which suggests that TGSS can learn characterizations related to the lipid solubility of molecules. In addition, it can also be found from Figure 5b that oxy acetonitrile, phenyl, 3,4,5-trihydroxy-6-(hydroxymethyl)oxan-2-yl, related to the water solubility of the molecule, are all clustered. Therefore, the TGSS model was able to effectively extract molecular property-related information.

## 3. Discussion

In this work, we explored the fusion of multiple models for molecular representation through generative self-supervised learning. TGSS, a triple generative self-supervised learning method, is proposed, which uses BiLSTM and Transformer through molecular sequences and GAT through 2D graphs for pre-training. Moreover, molecules are reconstructed by VAE between each model in pre-training. In downstream tasks, the trained models were fine-tuned and a feature fusion module was added to balance the weights between three molecular features. 

We experimentally validated the accuracy and generalization of the TGSS model using benchmark datasets from the fields of chemistry and biology, which indicates that pre-training with a large unlabeled dataset is effective for property prediction, since pre-training can enable the model to learn more molecular data and make up for the lack of labeled data. Meanwhile, by comparing it with other self-supervised learning methods, it was proven that our self-supervised strategy could extract molecular property-related representations more effectively, since this strategy fully combines multiple molecular features and more comprehensively obtains the information contained in the molecules. 

In addition, we verified the impact of pre-training weights, pre-training data volume, different model combinations, and molecular feature fusion on model performance through ablation experiments. By pre-training the model, the fitting speed and accuracy of the model in downstream tasks could be significantly accelerated. Different amounts of pre-training data also affect the performance of the model. The more pre-training data, the better the effect of the model. By using a combination of three models, the characteristics of different models can be fully exploited to improve the comprehensiveness of extracted molecular features. The added molecular feature fusion can effectively balance the proportions between different molecular features, and improve the performance of the final prediction. 

To validate the interpretability of the model, heat maps were generated by computing similarity coefficients, which revealed a high degree of consistency with the depiction of molecular structure in reality. It is demonstrated that the proposed model could extract key information from molecules.

## 4. Materials and Methods

### 4.1. Overview

**Figure 6:**
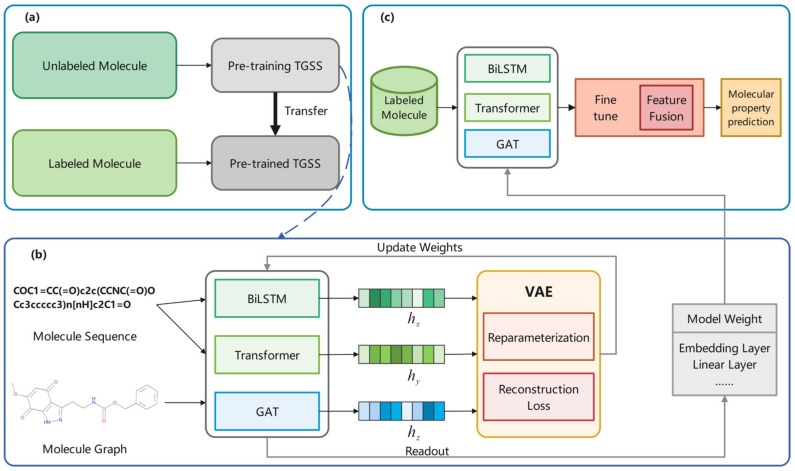
Overall framework. (**a**) TGSS framework. (**b**) Description of the generative self-supervised strategy in pre-training, the training model is updated according to reconstruction Loss. (**c**) Downstream task prediction.

This section presents the proposed triple generative self-supervised learning method based on molecular sequences and graph structures, which consists of two parts, a pre-training stage and a downstream task prediction stage, as shown in Figure 6a. Unlabeled molecules were used to train the TGSS model in pre-training, and the trained weights were transferred to the pre-trained TGSS model for molecular prediction.

In the pre-training part, the data used were all unlabeled, all of which came from a subset of the ChEMBL dataset, with a total of 430,000 molecules. In the encoder part, BiLSTM and Transformer were used to encode sequence data, and GAT was used to encode graph data. After obtaining the corresponding features, the VAE was used for generative self-supervised learning. As shown in Figure 6b, the input molecules were processed by three models to obtain $h_{x}$, $h_{y}$, $h_{z}$. These three features entered the VAE, where the reconstruction loss was calculated after reparameterization. The model weights were optimized based on the loss, and the weight with the best effect was used for downstream tasks. 

In the downstream task prediction, the data used were labeled data from MoleculeNet as shown in Figure 6c. The model was finetuned through the labeled data for prediction. Moreover, a feature fusion module was introduced to balance the proportions of each feature in the final output.

### 4.2. Pre-Training Models

In the pre-training stage, three models were utilized for training: BiLSTM, Transformer, and GAT, and the parameters of these models were transferred to downstream tasks for molecular property prediction.

There are two types of molecular input, which are molecular sequences (SMILES) and 2D molecular graphs. Sequence-based BiLSTM and Transformer are used to process SMILES to obtain the corresponding molecular features $h_{x}$ and $h_{y}$. Graph-based GAT is used to process 2D molecular graphs to obtain molecular features $h_{z}$. Each model will be introduced in the following sections.

#### 4.2.1. BiLSTM

**Figure 7:**
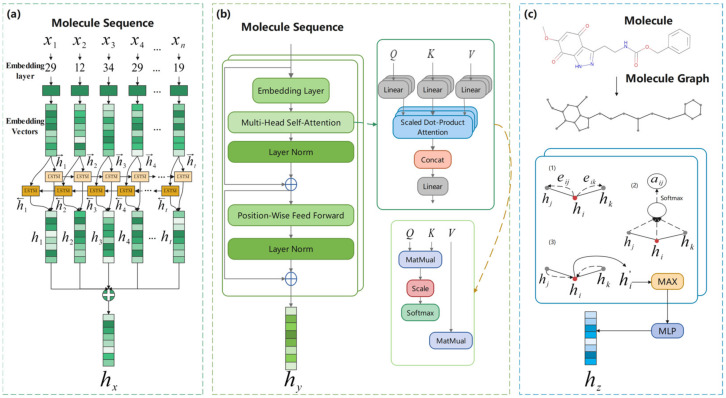
BiLSTM, Transformer and GAT models used in the TGSS model. (**a**) BiLSTM. (**b**) Transformer. (**c**) GAT.

BiLSTM, as an extension of the Recurrent Neural Network (RNN), addresses the challenges faced by RNN in learning long-term dependencies. The LSTM consists of three gate units: forget gate, input gate, and output gate. These gate units enable the model to extract features from the input data and keep this information for a long time. During the training process, the information is kept or discarded based on the weight value. Figure 7a shows the basic structure of BiLSTM, where $X=\left\{x_{1},x_{2},x_{3},\cdots ,x_{n}\right\}$ represents the elements in SMILES, $\left\{{\vec{h}}_{1},{\vec{h}}_{2},{\vec{h}}_{3},\cdots ,{\vec{h}}_{t}\right\}$ represents the hidden vector of the forward layer, and $\left\{{\overset{\leftarrow }{h}}_{1},{\overset{\leftarrow }{h}}_{2},{\overset{\leftarrow }{h}}_{3},\cdots ,{\overset{\leftarrow }{h}}_{t}\right\}$ represents the hidden vector of the backward layer. The input $\left\{x_{1},x_{2},x_{3},\cdots ,x_{n}\right\}$ are fed into the embedding layer to obtain the corresponding embedding vector, and the forward layer and the backward layer are used to obtain ${\vec{h}}_{t}$ and ${\overset{\leftarrow }{h}}_{t}$, respectively. These vectors are then combined to obtain the output vector $h_{x}$ of BiLSTM.

For an input $x_{t}$, the computation proceeds as follows:

(1) $$f_{t}=sigmoid\left(W_{f}\cdot \left[h_{t-1},x_{t}\right]+b_{f}\right)$$

(2) $$i_{t}=sigmoid\left(W_{i}\cdot \left[h_{t-1},x_{t}\right]+b_{i}\right)$$

(3) $${\tilde{C}}_{t}=tanh\left(W_{c}\cdot \left[h_{t-1},x_{t}\right]+b_{C}\right)$$

(4) $$C_{t}=f_{t}\;*\;C_{t-1}+i_{t}\;*\;{\tilde{C}}_{t}$$

(5) $$o_{t}=\sigma \left(W_{o}\cdot \left[h_{t-1},x_{t}\right]+b_{o}\right)$$

(6) $$h_{t}=o_{t}\;*\;tanh\left(C_{t}\right)$$

where $W_{f}$, $W_{i}$, $W_{c}$, $W_{o}$ are weight matrices, and $b_{f}$, $b_{i}$, $b_{C}$, $b_{o}$ are biases.

The BiLSTM utilizes two LSTMs with different directions. namely the forward layer and the backward layer, to process the input data. At time t, the forward layer calculates the hidden vector ${\vec{h}}_{t}$ at the current moment based on the previous hidden vector ${\vec{h}}_{t-1}$ and the embedding vector $X_{t}$; the backward layer calculates the hidden vector ${\overset{\leftarrow }{h}}_{t}$ based on ${\overset{\leftarrow }{h}}_{t-1}$ and the embedding vector $X_{t}$. Subsequently, ${\vec{h}}_{t}$ and ${\overset{\leftarrow }{h}}_{t}$ are combined to form the final hidden vector, which serves as the output of BiLSTM as follws:

(7) $$h_{x}=\left[{\vec{h}}_{t},{\overset{\leftarrow }{h}}_{t}\right]$$

#### 4.2.2. Transformer

The Transformer consists of a self-attention layer and a feed-forward neural network to capture the global dependencies between input and output through an attention mechanism. When processing a sequence, the RNN operates by sequentially processing words and passing the results to the next layer. However, when dealing with long sequences, the gradient tends to vanish or explode when words are distant from each other. Unlike RNN, the Transformer [48] tracks the relationship between words in the long text in both forward and backward directions through the attention mechanism. A detailed flowchart of Transformer is shown in Figure 7b.

First, an embedding layer is used to convert the input to $X_{embedding}\in R^{B\times S\times d}$, where B, S, and d represent the batch size, sequence length, and vector dimension, respectively. Subsequently, Q, K, V are obtained through linear transformation.

To address the problem of sequence prediction, the Transformer provides sequential information by adding position encoding $X_{pos}$ with the same dimensions as the input is obtained, which is combined with $X_{embedding}$ to obtain a new embedding as follows:

(8) $${X^{\prime }}_{embedding}=X_{embedding}+X_{pos}$$

Next, the self-attention mechanism is introduced to ensure the model attends on the more relevant characters as follows:

(9) $$Q={X^{\prime }}_{embedding}\times W_{Q}$$

(10) $$K={X^{\prime }}_{embedding}\times W_{K}$$

(11) $$V={X^{\prime }}_{embedding}\times W_{V}$$

where $W_{Q}$, $W_{K}$, and $W_{V}$ are trainable parameters, and then the attention matrix is calculated by ${QK}^{T}$ and weighted by *V*:

(12) $$Attention\left(Q,K,V\right)=softmax\left(\frac{{QK}^{T}}{\sqrt{d_{k}}}\right)V$$

where $d_{k}$ represents the number of columns in the Q, K matrix.

Then, the residual connection and layer normalization are implemented to obtain the $X_{hidden}$:

(13) $$X_{hidden}=Norm\left({X^{\prime }}_{embedding}+Attention\left(Q,K,V\right)\right)$$

The $X_{hidden}$ is used as the input to the Feed Forward Network, which contains two linear transformations to obtain the final output $h_{y}$ as the following equation:

(14) $$h_{y}=FFN\left(x\right)=max\left(0,X_{hidden}\cdot W_{1}+b_{1}\right)W_{2}+b_{2}$$

#### 4.2.3. GAT

Molecules can be represented as topological graphs by treating atoms as nodes and bonds as edges, which can be defined as $G=\left(V,E\right)$, where V denotes the set of nodes and E denotes the set of edges. A two-layer graph attention network [49] is used for node aggregation to obtain graph representations in TGSS. The processing flow of a molecule in GAT is shown in Figure 7c. First, the topological information is obtained from the molecular graph. The processing in GAT is divided into three steps.

The first step is to calculate the attention weights $e_{ij}$ and $e_{ik}$ of the central atom and the neighbor atoms through the following equation:

(15) $$e_{ij}=LeakyReLU\left(a^{T}\left[Wh_{i}||Wh_{j}\right]\right)$$

The second step is to normalize the weights to obtain $a_{ij}$, in which the $e_{ij}$ is fed into a softmax function for normalization.

(16) $$a_{ij}=softmax\left(e_{ij}\right)=\frac{exp\left(e_{ij}\right)}{\sum_{k\in N_{i}}exp\left(e_{ik}\right)}$$

Finally, the feature information of neighbor nodes are aggregated with the feature weight of its own node, through aggregating node weight information using the following equation:

(17) $$h_{i}^{\prime }=\sigma \left(\sum_{j\in N_{i}}a_{ij}Wh_{j}\right)$$

After obtaining the features of each atom i, max pooling and MLP are used to obtain the feature $h_{z}$.

(18) $$h_{z}=MLP\left(MAXPOOLING\left(h_{i}^{\prime }\right)\right)$$

### 4.3. Molecular Representation Reconstruction

**Figure 8:**
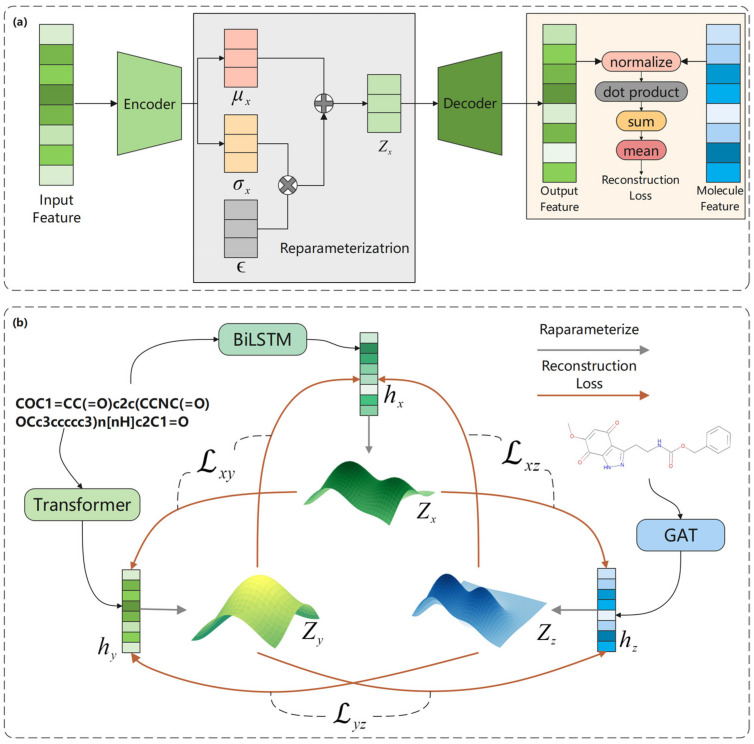
The process of molecular reconstruction loss calculation. (**a**) Calculation process of VAE. (**b**) Calculation process of molecular representation reconstruction loss. The loss between two molecular features is calculated by VAE. The three molecular features are first obtained from the corresponding latent vectors $z_{x}$, $z_{y}$, and $z_{z}$ through reparameterization of VAE. The reconstruction losses $L_{xy}$, $L_{xz}$, and $L_{yz}$ with the other two molecular features are obtained, respectively.

In the pre-training process, VAE is used to reconstruct the molecular features and calculate the reconstruction loss, as shown in Figure 8a. VAE consists of two parts: an encoder and a decoder. The encoder processes the input features to obtain mean $\mu_{x}$ and logarithm $\sigma_{x}$ which determine the latent vector $z_{x}$, and can be represented as follows:

(19) $$z_{x}=\mu_{x}+\sigma_{x}⊙\epsilon ,\;\;\epsilon ∼N\left(0,I\right)$$

In the decoder part, the reparameterization is implemented to calculate the latent vector $z_{x}$, and then the reconstructed features are output through the decoder. The reconstruction loss is obtained by calculating Mutual information (MI) between the two reconstructed features.

MI measures the nonlinear dependence between two random variables, and the larger the MI, the stronger the dependence between the variables. Unlike the correlation coefficient, MI is more general and determines the difference between the joint distribution of $p\left(x,y\right)$ and the product of the marginal distributions of $p\left(x\right)$ and $p\left(y\right)$. The standard expression of MI is calculated as follows, where $h_{x}$, $h_{y}$, and $h_{z}$ correspond to the feature of BiLSTM, Transformer, and GAT, respectively.

(20) $$I\left(h_{x};h_{y}\right)=E_{p\left(x,y\right)}\left[log\frac{p\left(x,y\right)}{p\left(x\right)p\left(y\right)}\right]$$

where $p\left(x,y\right)$ represents the joint probability distribution function of $h_{x}$ and $h_{y}$, while $p\left(x\right)$ and $p\left(y\right)$ represents the marginal probability distribution functions of $h_{x}$ and $h_{y}$, respectively.

It can be seen from the above equation that the greater the divergence of the product of $p\left(x,y\right)$ and $p\left(x\right)p\left(y\right)$, the stronger the correlation between x and y.

Therefore, our objective is to maximize the MI between any two features through the above models in order to obtain a more accurate representation, i.e., maximize $I\left(h_{x};h_{y}\right)$, $I\left(h_{x};h_{z}\right)$, $I\left(h_{y};h_{z}\right)$. In other words, it is used to minimize the difference between the reconstructed features and other features; that is, minimize $L_{xy}$, $L_{xz}$, and $L_{yz}$ in Figure 8b.

In this paper, we employed the variational lower bound to approximate the conditional log-likelihood term in (20). Specifically, when generating Transformer sequence features from the corresponding BiLSTM sequence features, we modeled the conditional likelihood $p\left(y|x\right)$ to obtain the lower bound of the conditional likelihood. Similarly, $p\left(z|x\right)$ represents the generation of GAT features from the BiLSTM sequence features, and $p\left(z|y\right)$ represents the generation of GAT graph features from Transformer sequence features. The calcuation of $p\left(y|x\right)$ is as follows:

(21) $$logp\left(y|x\right)\geq E_{q\left(z_{x}|x\right)}\left[logp\left(y|z_{x}\right)\right]-KL\left(q\left(z_{x}|x\right)||p\left(z_{x}\right)\right)$$

Likewise, the expression for $logp\left(x|y\right)$ is similar. The above objective function consists of conditional log-likelihood and Kullback–Leible (KL) divergence, which represent the reconstruction of a Transformer’s sequence features (y) from a sampled BiLSTM’s sequence features $\left(z_{x}\right)$. However, one challenge arises from the discrete nature of molecules, making them difficult to model in the molecular space.

Therefore, taking inspiration from Liu et al. [32], we implemented the reconstruction of the data space as a continuous representation space. For the reconstruction, we projected the latent vector $z_{x}$ onto the objective representation space. Then, the lower bound of the conditional likelihood could be calculated as follows:

(22) $$E_{q\left(z_{x}|x\right)}\left[logp\left(y|z_{x}\right)\right]=-E_{q\left(z_{x}|x\right)}\left[{\left\|q_{x}\left(z_{x}\right)-SGh_{y}\left(y\right)\right\|}_{2}^{2}\right]+C$$

where C is a constant, and the SG denotes the regularization operator used for optimizing the variational representation reconstruction.

Combining the above two equations with BiLSTM and Transformer as an example, the objective function of their loss can be calculated as follows:

(23) $$L_{xy}=\frac{1}{2}\cdot \left[E_{q\left(z_{x}|x\right)}\left[{\left\|q_{x}\left(z_{x}\right)-SGh_{y}\left(y\right)\right\|}^{2}\right]+E_{q\left(z_{y}|y\right)}\left[{\left\|q_{y}\left(z_{y}\right)-SGh_{x}\right\|}_{2}^{2}\right]\right]+\frac{\beta }{2}\;\cdot \left[KL\left(q\left(z_{x}|x\right)||p\left(z_{x}\right)\right)+KL\left(q\left(z_{y}|y\right)||p\left(z_{y}\right)\right)\right]$$

Similarly, the $L_{xz}$ between BiLSTM and GAT, as well as the $L_{yz}$ between Transformer and GAT, can also be calculated using the above equation. The final objective function is as follows:

(24) $$L=mean\left(L_{xy},L_{xz},L_{yz}\right)$$

### 4.4. Downstream Task with Hierarchical Elem-Feature Fusion

**Figure 9:**
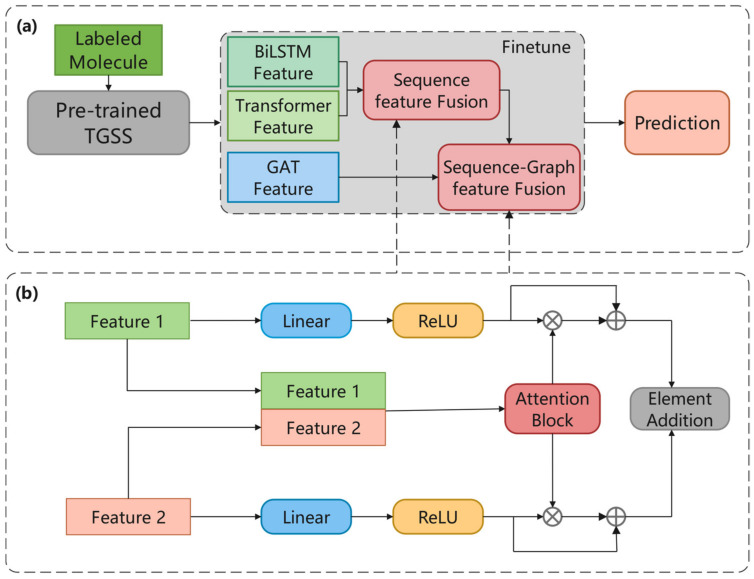
Downstream task with hierarchical elem-feature fusion. (**a**) The process of downstream task prediction process. (**b**) The process of molecular feature fusion.

The downstream task prediction stage includes three parts: model fine-tuning, hierarchical feature fusion, and molecular property prediction, as shown in Figure 9a. First, the model reloads the pre-training weights and fine-tunes them according to the input labelled data. In the fine-tuning stage, three models including BiLSTM, Transformer, and GAT output features, and the hierarchical feature fusion is performed, respectively, sequence feature fusion and sequence-graph feature fusion. The final output is used for molecular property prediction.

Since three models are used to obtain three molecular features, just concatenating them directly cannot fully explore the information hidden in the features and find the important part of each feature. Inspired by Hua et al. [50], we combined the sequence and structural features and balanced the weights of different features, and the two same feature fusion models were adopted in the TGSS model, as shown in Figure 9b.

First, the sequence features of molecules are fused by combining the BiLSTM feature and the Transformer feature. The weights of the two features are obtained through the attention module shown in Equation (25):

(25) $$W_{attn}=\sigma \left({CNN}_{2D}\left(Concat\left(h_{x},h_{y}\right)\right)\right)$$

where $h_{x}\in R^{N\times d}$ and $h_{y}\in R^{N\times d}$ and $h_{x}$ and $h_{y}$ denote the BiLSTM feature and Transformer feature, respectively. Concat is the concatenating operation, and the features are extracted from the concatenated feature map by 2D convolution operation ${CNN}_{2D}$. The σ is a Sigmoid used to normalize the convolved features to obtain the attention weight matrix $W_{attn}$ of $h_{x}$. The (1 − $W_{attn}$) is defiend as the attention weight matrix of $h_{y},$ correspondingly. After connecting the residuals of the two features separately, the combined feature $h_{f}$ is obtained by Equation (26):

(26) $$h_{f}=FC\left(h_{x}\right)\;*\;W_{attn}+h_{x}+FC\left(h_{y}\right)\;*\;\left(1-W_{attn}\right)+h_{y}$$

where FC denotes the Linear layer and the ReLU layer, and ∗ denotes the element dot product.

Following the above method, the sequence feature $h_{f}$ is fused with the graph feature $h_{z}$ to obtain the final feature $h_{f}$. After hierarchical fusion, the output $h_{f}$ is robust and fully combines the sequence and graph information of molecules, which can be used for more effective molecular predictions.

## 5. Conclusions

Molecular property prediction is an important task in molecular design. Deep learning methods are used to effectively extract molecular features, thereby reducing the time and costs required. We use three models to extract features from different dimensions to ensure that as much molecular information is retained as possible. In pre-training, a generative self-supervised strategy was adopted. Among them, VAE was utilized to calculate the reconstruction molecule loss and optimize the model based on the reconstruction loss. It turns out that generative self-supervised learning can provide great help for molecular sequence representation and graph representation.

Our current research only focused on the 1D and 2D information of molecules. In the future, 3D information could be considered as useful information which may be introduced for in-depth research. Additionally, we would like to utilize larger pre-trained datasets to improve the comprehensiveness of the model. Through our work, we hope we can make contributions to molecular property prediction.

## Figures and Tables

**Table 1:** The details of the MoleculeNet Datasets.

Dataset	Task	Task Type	#Molecule	Splits	Metric
FreeSolv	1	Regression	642	Random	RMSE
ESOL	1	Regression	1128	Random	RMSE
Lipophilicity	1	Regression	4200	Random	RMSE
BACE	1	Classification	1513	Scaffold	ROC-AUC
BBBP	1	Classification	2039	Scaffold	ROC-AUC
HIV	1	Classification	41127	Scaffold	ROC-AUC
Tox21	12	Classification	7831	Scaffold	ROC-AUC
SIDER	27	Classification	1427	Scaffold	ROC-AUC

**Table 2:** The ROC-AUC values of various approaches in classification tasks. Higher values mean better results.

	Dataset	BACE	BBBP	HIV	Tox21	SIDER
Supervised learning	D-MPNN	0.809 (0.006)	0.710 (0.003)	0.771 (0.005)	0.759 (0.007)	0.570 (0.007)
	AttentionFP	0.784 (0.022)	0.643 (0.018)	0.757 (0.014)	0.761 (0.005)	0.606 (0.032)
	MSSGAT	0.881	0.726	0.787	-	0.617
Self-Supervised learning	MolCLR	0.828 (0.007)	0.733 (0.010)	0.774 (0.006)	0.741 (0.053)	0.612 (0.036)
	GraphCL	0.754 (0.014)	0.697 (0.007)	0.698 (0.027)	0.739 (0.007)	0.605 (0.009)
	HierMRL	**0.877 (0.017)**	0.745 (0.016)	0.782 (0.011)	**0.792 (0.006)**	0.686 (0.011)
	GraphLoG	0.835 (0.012)	0.657 (0.014)	0.778 (0.008)	0.757 (0.006)	0.612 (0.011)
	GraphMVP	0.768 (0.011)	0.685 (0.002)	0.748 (0.014)	0.745 (0.004)	0.623 (0.016)
	GraphMAE	0.831 (0.009)	0.720 (0.006)	0.772 (0.010)	0.755 (0.006)	0.603 (0.011)
TGSS		0.810(0.004)	**0.790 (0.068)**	**0.789 (0.041)**	0.754 (0.005)	**0.721 (0.004)**

Note: The best results are shown in bold. Standard deviations are in brackets.

**Table 3:** The RMSE values of various approaches in regression tasks. Lower values mean better results.

	Dataset	FreeSolv	ESOL	Lipophilicity
Supervised learning	D-MPNN	2.082 (0.082)	1.050 (0.008)	0.683 (0.016)
	DimeNet	2.094 (0.118)	0.878 (0.023)	0.727 (0.019)
	DLF-MFF	1.849	0.747	0.772
Self-Supervised learning	KEMPNN	1.188 (0.158)	0.703 (0.024)	**0.563 (0.011)**
	MolPMoFiT	1.197 (0.127)	-	0.565 (0.037)
	MolBERT	1.523 (0.660)	**0.552 (0.070)**	0.602 (0.010)
	FP-BERT	1.140 (0.006)	0.670 (0.004)	0.660 (0.002)
	SMILES Transformer	1.650	0.720	0.921
TGSS		**0.960 (0.065)**	0.645 (0.075)	0.652 (0.009)

Note: The best results are shown in bold. Standard deviations are in brackets.

## Data Availability

The code and data are provided at https://github.com/853683892/TGSS (accessed on 11 December 2023).

## References

[1] Gervasoni S., Manelfi C., Adobati S., Talarico C., Biswas A.D., Pedretti A., Vistoli G., Beccari A.R. (2023). Target Prediction by Multiple Virtual Screenings: Analyzing the SARS-CoV-2 Phenotypic Screening by the Docking Simulations Submitted to the MEDIATE Initiative. Int. J. Mol. Sci..

[2] Moschovou K., Antoniou M., Chontzopoulou E., Papavasileiou K.D., Melagraki G., Afantitis A., Mavromoustakos T. (2023). Exploring the Binding Effects of Natural Products and Antihypertensive Drugs on SARS-CoV-2: An in Silico Investigation of Main Protease and Spike Protein. Int. J. Mol. Sci..

[3] Blanco-Gonzalez A., Cabezon A., Seco-Gonzalez A., Conde-Torres D., Antelo-Riveiro P., Pineiro A., Garcia-Fandino R. (2023). The Role of Ai in Drug Discovery: Challenges, Opportunities, and Strategies. Pharmaceuticals.

[4] Dara S., Dhamercherla S., Jadav S.S., Babu C.M., Ahsan M.J. (2022). Machine Learning in Drug Discovery: A Review. Artif. Intell. Rev..

[5] Aliev T.A., Belyaev V.E., Pomytkina A.V., Nesterov P.V., Shityakov S., Sadovnichii R.V., Novikov A.S., Orlova O.Y., Masalovich M.S., Skorb E.V. (2023). Electrochemical Sensor to Detect Antibiotics in Milk Based on Machine Learning Algorithms. ACS Appl. Mater. Interfaces.

[6] Wang X., Liu D., Zhu J., Rodriguez-Paton A., Song T. (2021). CSConv2d: A 2-D Structural Convolution Neural Network with a Channel and Spatial Attention Mechanism for Protein-Ligand Binding Affinity Prediction. Biomolecules.

[7] Xu L., Pan S., Xia L., Li Z. (2023). Molecular Property Prediction by Combining LSTM and GAT. Biomolecules.

[8] Xia L., Xu L., Pan S., Niu D., Zhang B., Li Z. (2023). Drug-Target Binding Affinity Prediction Using Message Passing Neural Network and Self Supervised Learning. BMC Genom..

[9] Pan S., Xia L., Xu L., Li Z. (2023). SubMDTA: Drug Target Affinity Prediction Based on Substructure Extraction and Multi-Scale Features. BMC Bioinform..

[10] Li X., Han P., Wang G., Chen W., Wang S., Song T. (2022). SDNN-PPI: Self-Attention with Deep Neural Network Effect on Protein-Protein Interaction Prediction. BMC Genom..

[11] Rogers D., Hahn M. (2010). Extended-Connectivity Fingerprints. J. Chem. Inf. Model..

[12] Durant J.L., Leland B.A., Henry D.R., Nourse J.G. (2002). Reoptimization of MDL Keys for Use in Drug Discovery. J. Chem. Inf. Comput. Sci..

[13] Wieder O., Kohlbacher S., Kuenemann M., Garon A., Ducrot P., Seidel T., Langer T. (2020). A Compact Review of Molecular Property Prediction with Graph Neural Networks. Drug Discov. Today Technol..

[14] Hou Y., Wang S., Bai B., Chan H.C.S., Yuan S. (2022). Accurate Physical Property Predictions via Deep Learning. Molecules.

[15] Honda S., Shi S., Ueda H.R. (2019). SMILES Transformer: Pre-Trained Molecular Fingerprint for Low Data Drug Discovery. arXiv.

[16] Ma H., Bian Y., Rong Y., Huang W., Xu T., Xie W., Ye G., Huang J. (2020). Multi-View Graph Neural Networks for Molecular Property Prediction. arXiv.

[17] Jiang S., Balaprakash P. (2020). Graph Neural Network Architecture Search for Molecular Property Prediction. Proceedings of the 2020 IEEE International Conference on Big Data (Big Data).

[18] Chen J., Zheng S., Song Y., Rao J., Yang Y. (2021). Learning Attributed Graph Representations with Communicative Message Passing Transformer. arXiv.

[19] Song Y., Zheng S., Niu Z., Fu Z., Lu Y., Yang Y. (2020). Communicative Representation Learning on Attributed Molecular Graphs. Proceedings of the Twenty-Ninth International Joint Conference on Artificial Intelligence.

[20] Shahab M., Zheng G., Khan A., Wei D., Novikov A.S. (2023). Machine Learning-Based Virtual Screening and Molecular Simulation Approaches Identified Novel Potential Inhibitors for Cancer Therapy. Biomedicines.

[21] Zhao X., Huang L., Nie J., Wei Z. (2024). Towards Adaptive Multi-Scale Intermediate Domain via Progressive Training for Unsupervised Domain Adaptation. IEEE Trans. Multimed..

[22] Liu X., Zhang F., Hou Z., Mian L., Wang Z., Zhang J., Tang J. (2021). Self-Supervised Learning: Generative or Contrastive. IEEE Trans. Knowl. Data Eng..

[23] Wang J., Guan J., Zhou S. (2023). Molecular Property Prediction by Contrastive Learning with Attention-Guided Positive Sample Selection. Bioinformatics.

[24] Cao H., Huang L., Nie J., Wei Z. (2024). Unsupervised Deep Hashing with Fine-Grained Similarity-Preserving Contrastive Learning for Image Retrieval. IEEE Trans. Circuits Syst. Video Technol..

[25] Brown T.B., Mann B., Ryder N., Subbiah M., Kaplan J., Dhariwal P., Neelakantan A., Shyam P., Sastry G., Askell A. (2020). Language Models Are Few-Shot Learners. Adv. Neural Inf. Process. Syst..

[26] Devlin J., Chang M.-W., Lee K., Toutanova K. (2019). BERT: Pre-Training of Deep Bidirectional Transformers for Language Understanding. arXiv.

[27] Wen N., Liu G., Zhang J., Zhang R., Fu Y., Han X. (2022). A Fingerprints Based Molecular Property Prediction Method Using the BERT Model. J. Cheminform..

[28] Qiu J., Chen Q., Dong Y., Zhang J., Yang H., Ding M., Wang K., Tang J. (2020). GCC: Graph Contrastive Coding for Graph Neural Network Pre-Training. Proceedings of the 26th ACM SIGKDD International Conference on Knowledge Discovery & Data Mining.

[29] Li H., Zhang R., Min Y., Ma D., Zhao D., Zeng J. (2023). A Knowledge-Guided Pre-Training Framework for Improving Molecular Representation Learning. Nat. Commun..

[30] Zhang S., Hu Z., Subramonian A., Sun Y. (2024). Motif-Driven Contrastive Learning of Graph Representations. IEEE Trans. Knowl. Data Eng..

[31] Zang X., Zhao X., Tang B. (2023). Hierarchical Molecular Graph Self-Supervised Learning for Property Prediction. Commun. Chem..

[32] Liu S., Wang H., Liu W., Lasenby J., Guo H., Tang J. (2022). Pre-Training Molecular Graph Representation with 3D Geometry. arXiv.

[33] Zhu J., Xia Y., Wu L., Xie S., Zhou W., Qin T., Li H., Liu T.-Y. (2023). Dual-View Molecular Pre-Training. Proceedings of the 29th ACM SIGKDD Conference on Knowledge Discovery and Data Mining.

[34] Wu Z., Ramsundar B., Feinberg E.N., Gomes J., Geniesse C., Pappu A.S., Leswing K., Pande V. (2018). MoleculeNet: A Benchmark for Molecular Machine Learning. Chem. Sci..

[35] Yang K., Swanson K., Jin W., Coley C., Eiden P., Gao H., Guzman-Perez A., Hopper T., Kelley B., Mathea M. (2019). Analyzing Learned Molecular Representations for Property Prediction. J. Chem. Inf. Model..

[36] Wang Y., Wang J., Cao Z., Farimani A.B. (2022). Molecular Contrastive Learning of Representations via Graph Neural Networks. Nat. Mach. Intell..

[37] You Y., Chen T., Sui Y., Chen T., Wang Z., Shen Y. (2020). Graph Contrastive Learning with Augmentations. Adv. Neural Inf. Process. Syst..

[38] Liu M., Yang Y., Gong X., Liu L., Liu Q. (2022). HierMRL: Hierarchical Structure-Aware Molecular Representation Learning for Property Prediction. Proceedings of the 2022 IEEE International Conference on Bioinformatics and Biomedicine (BIBM).

[39] Xu M., Wang H., Ni B., Guo H., Tang J. Self-Supervised Graph-Level Representation Learning with Local and Global Structure. Proceedings of the International Conference on Machine Learning.

[40] Hou Z. GraphMAE: Self-Supervised Masked Graph Autoencoders. Proceedings of the 28th ACM SIGKDD Conference on Knowledge Discovery and Data Mining.

[41] Fang Y., Zhang Q., Zhang N., Chen Z., Zhuang X., Shao X., Fan X., Chen H. (2023). Knowledge Graph-Enhanced Molecular Contrastive Learning with Functional Prompt. Nat. Mach. Intell..

[42] Li X., Fourches D. (2020). Inductive Transfer Learning for Molecular Activity Prediction: Next-Gen QSAR Models with MolPMoFiT. J. Cheminform..

[43] Fabian B., Edlich T., Gaspar H., Segler M., Meyers J., Fiscato M., Ahmed M. (2020). Molecular Representation Learning with Language Models and Domain-Relevant Auxiliary Tasks. arXiv.

[44] Gasteiger J., Groß J., Günnemann S. (2022). Directional Message Passing for Molecular Graphs. arXiv.

[45] Xiong Z., Wang D., Liu X., Zhong F., Wan X., Li X., Li Z., Luo X., Chen K., Jiang H. (2020). Pushing the Boundaries of Molecular Representation for Drug Discovery with the Graph Attention Mechanism. J. Med. Chem..

[46] Ma M., Lei X. (2024). A Deep Learning Framework for Predicting Molecular Property Based on Multi-Type Features Fusion. Comput. Biol. Med..

[47] Ye X., Guan Q., Luo W., Fang L., Lai Z.-R., Wang J. (2022). Molecular Substructure Graph Attention Network for Molecular Property Identification in Drug Discovery. Pattern Recognit..

[48] Vaswani A., Shazeer N., Parmar N., Uszkoreit J., Jones L., Gomez A.N., Kaiser Ł., Polosukhin I. Attention Is All You Need. Proceedings of the 31st Conference on Neural Information Processing Systems (NIPS 2017).

[49] Veličković P., Cucurull G., Casanova A., Romero A., Liò P., Bengio Y. (2018). Graph Attention Networks. arXiv.

[50] Hua Y., Song X., Feng Z., Wu X. (2023). MFR-DTA: A Multi-Functional and Robust Model for Predicting Drug–Target Binding Affinity and Region. Bioinformatics.

